# Yuquan pill enhance the effect of Western medicine in treatment diabetic nephropathy: A protocol for systematic review and meta-analysis

**DOI:** 10.1097/MD.0000000000027555

**Published:** 2021-10-22

**Authors:** Le Liu, Ye Zhang, Zhiyue Zhu, Ziyang Yu, Pengjie Bao, Zheng Nan

**Affiliations:** a Changchun University of Traditional Chinese Medicine, China; b The Affiliated Hospital of Changchun University of Chinese Medicine, Changchun, China.

**Keywords:** diabetic nephropathy, systematic evaluation, traditional Chinese medicine, Yuquan pill

## Abstract

**Background:**

Diabetic nephropathy is glomerular sclerosis caused by diabetic microvascular disease, which is one of the most serious complications of diabetes. At present, the traditional treatment of diabetic nephropathy is mainly based on the control of blood pressure, blood sugar, blood lipids, and other basic treatments using angiotensin converting enzyme inhibitor/angiotensin receptor blocker, the clinical reports of relying solely on angiotensin converting enzyme inhibitor/ angiotensin receptor blocker to delay the course of diabetic nephropathy are not optimistic. Yuquan pill (YQP), a classic traditional Chinese medicine prescription, has clinical reports that it can effectively assist in the treatment of diabetic nephropathy with minimal side effects. However, there has been no systematic review of the YQP in the treatment of diabetic nephropathy. This article systematically evaluated the effectiveness and safety of YQP clinical applications.

**Methods:**

The database search include seven databases, including PubMed, Embase, Cochrane Library, Chinese Biomedical Literature Database, Chinese National Knowledge Infrastructure, Chinese Scientific Journal Database, and Wanfang database. The search date was set as a randomized controlled trial from the establishment of the database to April 21, 2021. The main outcome indicators include urinealbumin excretion rate, serum creatinine, urea nitrogen, Total effective rate. The analysis software uses Stata 15.

**Results:**

This study will analyze multiple outcome indicators such as clinical efficacy, urinary albumin excretion rate, blood creatinine value, urea nitrogen, and symptom scores. Provides a the latest evidence for YQP in the treatment of diabetic nephropathy (DN).

**Conclusion:**

The results of this study will provide evidence for the efficacy of YQP in the treatment of DN.

**INPLASY Registration number:**

INPLASY202150030.

## 1 Introduction

Diabetic nephropathy (DN) is a disease characterized by renal dysfunction and structural changes caused by the chronic microvascular disease of diabetes. Diabetic kidney disease (DKD) manifests insidiously in the early onset, develops rapidly, and cannot be reversed.^[1]^ In addition, clinical symptoms caused by aggravation of the disease are even more troublesome for patients, which severely reduces the quality of life of patients; therefore, it is particularly important to help delay their disease, reduce their chances of entering end-stage renal disease, and reduce their symptoms.^[2]^ Currently, modern medicine mainly focuses on anti-inflammatory, anti-oxidant, anti-fibrosis, and antagonistic endothelin receptors for DKD treatment.^[3]^ However, the clinical efficacy of Western medicine alone for DKD patients is not very satisfactory. In recent years, traditional Chinese medicine has achieved good clinical efficacy for DKD treatment with its unique advantages, starting from the overall concept and syndrome differentiation and treatment, especially in helping the patient has an obvious effect in reducing symptoms and has certain application prospects.

Yuquan pills (YQP) originated from “Renzhai Zhizhi, written by Yangrenzhai during the Song Dynasty. It is an ancient prescription for the treatment of diminished thirst. It has the effects of replenishing qi and nourishing yin, promoting body fluid and quenching thirst. Modern studies have found that Astragalus, Ophiopogon, Pueraria lobata, Shengdi, Licorice and other active ingredients in YQPs all have the effect of reducing urine protein and protecting kidney function.^[4,5]^ Studies have shown that YQP can significantly reduce DKD The level of TNF-α and IL-1 in patients has the ability to reduce the inflammation of DKD It can protect kidney function and reduce kidney damage.^[6]^ Therefore, the purpose of this study is to systematically review and analyze the existing literature and evaluate the effectiveness and safety of YQP in the treatment of diabetic nephropathy.

## 2 Methods

### 2.1 Protocol and registration

The agreement has been registered on the Open Science Framework (INPLASY) platform (https://inplasy.com/), registration number: INPLASY202150030. This program is the preferred reporting project based on the Guidelines for the Systematic Review and Meta-Analysis Program,^[7]^ and the final report will be in line with PRISMA's recommendations on the extended statement of the systematic review report included in the meta-analysis of medical interventions.^[8]^

### 2.2 Type of study

The included literature should be a clinical randomized controlled trial of YQP adjuvant treatment of diabetic nephropathy, the language of publication is Chinese or English, and the time and use of blinding are not limited.

### 2.3 Type of participant

According to the 2009 ADA Diabetes Diagnostic Criteria^[9]^ and Mogensen staging, patients who meet the diagnostic criteria of diabetic nephropathy, and except for acute metabolic diseases with diabetes, diabetic ketoacidosis, urinary tract infections, and severe heart, lung, and liver diseases.^[10]^ There are no requirements for gender, race, and age.

### 2.4 Intervention

The intervention method of the treatment group was YQP combined with Western medicine. The control group was treated with conventional Western medicine and there are no requirements for medication time, medication frequency, and drug dosage form.

### 2.5 Outcome indicators

1. Primary outcomes: urine albumin excretion rate, serum creatinine, urea nitrogen, Total effective rate.
2. Secondary outcomes: 24 hours urine protein quantification, fasting blood glucose, postprandial blood glucose, glycosylated hemoglobin, as well as TCM Syndrome Score Scale, etc.

### 2.6 Search strategy

#### 2.6.1 Electronic searches

The selection of the time for inclusion of the literature will be selected from the establishment of each database to April 21, 2021, by searching PubMed, Embase, Cochrane Library, Chinese Biomedical Literature Database, Chinese National Knowledge Infrastructure, Chinese Scientific Journal Database, Wanfang database, and other seven databases. Keywords include “Yuquan Pill,” “diabetic nephropathy,” and so on. Specific search terms in Table 1.

**Table 1:** Search strategy of the PubMed.

Number	Search terms
#1	Diabetic nephropathy [Mesh]
#2	Diabetic nephropathy[Title/Abstract] OR diabetic nephropathies[Title/Abstract] OR diabetic kidney disease[Title/Abstract] OR diabetic nephrosis [Title/Abstract] OR renal diabetes[Title/Abstract]
#3	#1 OR #2
#4	Yuquan Pill[Title/Abstract]
#5	Yuquan wan[Title/Abstract]
#6	#4 AND #5
#7	randomized controlled trial[Publication Type]
#8	controlled clinical trial[Publication Type]
#9	randomized[Title/Abstract]
#10	randomly[Title/Abstract]
#11	#10 OR #11 OR #12 OR #13
#12	#3 AND #6 AND #11

### 2.7 Data collection and analysis

#### 2.7.1 Included literature screening

**Figure 1:**
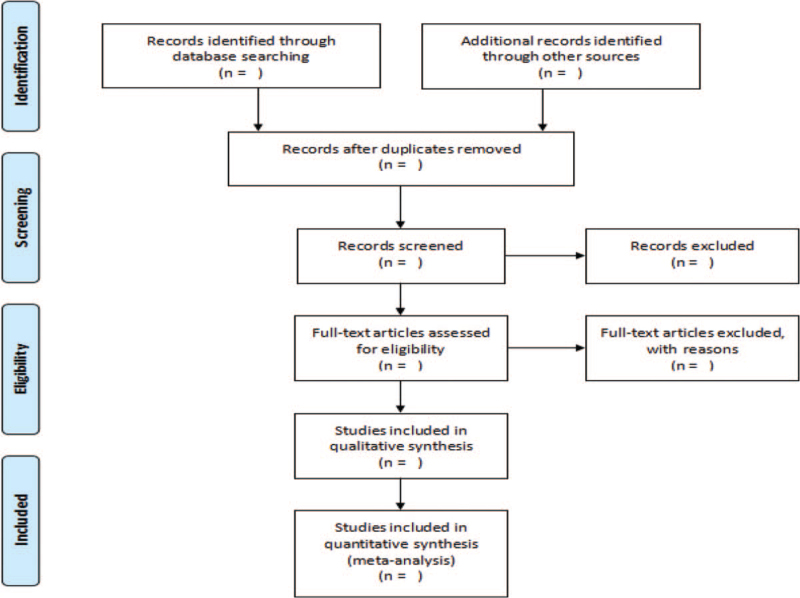
PRISMA flow diagram of the study selection process.

Two researchers (Le Liu and Ye Zhang) conducted a literature search. After retrieval, the literature was imported into Endnote X9 software for review. After deleting duplicate literature, read the abstract for preliminary screening, and download all literature after excluding those that obviously did not meet the inclusion criteria. The full text is carefully read, and further review. Finally, the documents that meet the inclusion criteria are screened out, and the data extraction table has been designed for data extraction, and 2 researchers conduct cross-checking. If the results are different, we will ask the third researcher (Nan Zheng) to assist in the judgment. The specific process is illustrated in Fig. 1.

#### 2.7.2 Research data extraction

Two researchers (Zhiyue Zhu and Ziyang Yu) carried out data extraction, using the same standard for data extraction, including the basic information of the included study, the characteristics of the included patients, intervention methods, and outcome indicators. If there are objections to data extraction, the third researcher (Pengjie Bao) will make the final judgment. If we find incomplete information and omissions in the included literature, we will conduct a specific analysis to review the impact of missing data on the results of the meta-analysis.

#### 2.7.3 Evidence quality evaluation

Referring to the quality evaluation standards recommended by the Cochrane Collaboration Network for quality evaluation, mainly include the random sequence generation method, whether allocation concealment is used, whether the subject and the intervention provider are blinded, whether the result evaluator is blind, whether the result data is complete, and whether selective results reporting and other sources of bias.^[11]^ The Quality evaluation is carried out by 2 researchers (LL and YZ), and the evaluation criteria include 3 categories (low risk, high risk, and unclear). If the quality audit is different, it will be decided through discussion with a third researcher (ZN). In all items, the answer “yes” means that there is a low risk of bias, the answer “no” is a high risk of bias, and the answer “unknown” means that it is uncertain whether there is a risk of bias.

#### 2.7.4 Data synthesis

This study will use The RevMan 5.0 software (version 5.3) and Stata 15.0 software provided by Cochrane for meta-analysis. The effect index was expressed as odds ratio (OR) and 95% confidence interval (95% CI). If there is statistical homogeneity (*I*^*2*^ < 50%) between the studies, the fixed-effect model is used. When the heterogeneity between the research results is significant (*I*^*2*^ ≥ 50%), sublayer analysis is needed to find the reason for the heterogeneity. If the heterogeneity is too large or the source of the heterogeneity is unknown, use qualitative heterogeneity. When there are many influencing factors and the stratification method is not suitable, meta-regression analysis is required. When the number of articles included in the analysis of effect indicators is ≥10, a funnel chart was used to analyze the risk of publication bias. When the funnel chart is obviously asymmetric, it indicates a publication bias.

#### 2.7.5 Assessment of heterogeneity and sensitivity analysis

*I*^*2*^ and Chi-Squared statistics were used to assess the statistical heterogeneity between studies. If *I*^*2*^ is between 50% and 100%, there is statistical heterogeneity, and we will use a random-effects model to analyze the data. If the heterogeneity test is not significant (*I*^*2*^ ≤ 50%), the fixed-effects model is used. In addition, due to differences in heterogeneity, we will conduct subgroup or sensitivity analysis to find potential causes and sensitivity analysis is used to eliminate studies with high risk of bias or incomplete data and the impact of outliers.

#### 2.7.6 Subgroup analysis

When there is disagreement in the results, a subgroup analysis needs to be carried out for different reasons. Heterogeneity is mainly manifested in many aspects such as race, sex, age, drug formulations, different forms of intervention, treatment time, and drug dosages.

#### 2.7.7 Grading the quality of the evidence

Evaluate the reliability of evidence, assign and evaluate grades, and systematically assess the quality of evidence. The quality of evidence will be divided into 4 grades: high, medium, low, and very low.^[12]^

#### 2.7.8 Ethics and dissemination

Our goal is to publish the results in a peer-reviewed journal. Because this study is a systematic review, there is no need for personal data for each patient. Approval from the institutional review board and approval of the ethics committee are also not required.

## 3 Discussions

With the continuous improvement of people's living standards, the incidence of diabetic nephropathy is increasing every year, and there are increasing numbers of patients with diabetic nephropathy.^[2]^ At the same time, the age of diabetic kidney disease is gradually decreasing.^[13]^ Diabetic nephropathy is the most important end-stage renal disease. The culprit is that treatment is currently restricted in many ways.^[14]^ The prevention and treatment of diabetic nephropathy has always been a direction that clinicians are exploring.^[15]^ A number of studies have shown that YQPs can effectively assist in the treatment of diabetic nephropathy, which can reduce proteinuria and improve renal function in patients.^[16]^ In addition, studies have shown that YQPs combined with Western medicine can effectively improve the renal function of patients with diabetic nephropathy and reduce inflammation and insulin resistance.^[17]^ However, there has been no systematic review of its effectiveness and safety. This systematic review will first summarize the current evidence on the effectiveness and safety of YQP in the treatment of DN. This study will be helpful for the treatment of diabetic nephropathy.

## Author contributions

**Conceptualization:** Le Liu.

**Data curation:** Ye Zhang, Zhiyue Zhu.

**Funding acquisition:** Zheng Nan.

**Investigation:** Ziyang Yu.

**Methodology:** Pengjie Bao.

**Project administration:** Zheng Nan.

**Resources:** Zhiyue Zhu.

**Supervision:** Ziyang Yu.

**Validation:** Ye Zhang.

**Writing – original draft:** Le Liu.

**Writing – review & editing:** Le Liu.
